# Interactions Between Thiamethoxam and *Deformed Wing Virus* Can Drastically Impair Flight Behavior of Honey Bees

**DOI:** 10.3389/fmicb.2020.00766

**Published:** 2020-04-30

**Authors:** Marianne Coulon, Anne Dalmon, Gennaro Di Prisco, Alberto Prado, Florine Arban, Eric Dubois, Magali Ribière-Chabert, Cedric Alaux, Richard Thiéry, Yves Le Conte

**Affiliations:** ^1^INRAE, UR 406 Abeilles et Environnement, Site Agroparc, Avignon, France; ^2^ANSES Sophia Antipolis, Unit of Honey bee Pathology, Sophia Antipolis, France; ^3^CREA-AA, Research Centre for Agriculture and Environment, Council for Agricultural Research and Economics, Bologna, Italy; ^4^Department of Agriculture, University of Naples “Federico II”, Portici, Italy; ^5^Escuela Nacional de Estudios Superiores Juriquilla, UNAM, Juriquilla, Mexico

**Keywords:** neonicotinoid, pesticide, synergy, pathogen, colony collapse, sub-lethal effects, foraging

## Abstract

Exposure to multiple stress factors is believed to contribute to honey bee colony decline. However, little is known about how co-exposure to stress factors can alter the survival and behavior of free-living honey bees in colony conditions. We therefore studied the potential interaction between a neonicotinoid pesticide, thiamethoxam, and a highly prevalent honey bee pathogen, *Deformed wing virus* (DWV). For this purpose, tagged bees were exposed to DWV by feeding or injection, and/or to field-relevant doses of thiamethoxam, then left in colonies equipped with optical bee counters to monitor flight activity. DWV loads and the expression of immune genes were quantified. A reduction in vitellogenin expression level was observed in DWV-injected bees and was associated with precocious onset of foraging. Combined exposure to DWV and thiamethoxam did not result in higher DWV loads compared to bees only exposed to DWV, but induced precocious foraging, increased the risk of not returning to the hive after the first flight, and decreased survival when compared to single stress exposures. We therefore provided the first evidence for deleterious interactions between DWV and thiamethoxam in natural conditions.

## Introduction

Heavy losses of honey bee colonies in the Northern hemisphere have been documented since the beginning of the 21st century (Lee et al., 2015). It is crucial to study such losses as it is known that 84% of the 264 most important crops in Europe depend on, at least to some extent, animal pollination (Williams, 1994). There is now a scientific consensus on the fact that honey bee colony losses are the result of multifactorial causes, including a decrease in floral resource availability, the spread of pathogens and pesticide use (Goulson et al., 2015).

Honey bee colonies are frequently co-exposed to several pesticides and their corresponding metabolites, even when foraging in grasslands or non-agricultural areas (Hladik et al., 2016; Long and Krupke, 2016; Alburaki et al., 2018). Indeed, pesticides are carried to the hive via contaminated pollen and nectar by forager bees. For instance, neonicotinoids have increasingly been used as insecticides for a wide range of crops for the past 20 years (Simon-Delso et al., 2015), which has resulted in frequent detection in honey bee hives (Lawrence et al., 2016) and hive products like honey (Jones and Turnbull, 2016; Mitchell et al., 2017). Despite EU policy measures for banning neonicotinoids they still persist in the environment (Wintermantel et al., 2020).

In addition, the high density of individuals inside the honey bee colony facilitates pathogen transmission. Honey bee colonies are favorable to pathogens due to the high concentration of individuals and stored food in the colony (Schmid-Hempel, 1998). Therefore, honey bees can often be co-exposed to stress factors, like pathogens and pesticides (Mullin et al., 2010; vanEngelsdorp and Meixner, 2010; Cornman et al., 2012; Simon-Delso et al., 2014). Interactions between pesticides and pathogens have been reviewed by Poquet et al. (2016). Specifically, pesticides can interact with bee viruses. DeGrandi-Hoffman et al. (2013) showed that the number of nurse bees contaminated with *Black queen cell virus* (BQCV) increased significantly when they were fed with pollen containing a mix of the insecticide chlorpyrifos (organophosphates) and a commercial fungicide solution. In the field, Alburaki et al. (2015) found higher BQCV loads in colonies located in treated corn fields grown from neonicotinoid-coated seeds but a subsequent study did not confirm this result (Osterman et al., 2019). Combined exposure to sublethal doses of the neonicotinoid thiacloprid and BQCV was also found to significantly increase mortality and BQCV viral loads in honey bee larvae (Doublet et al., 2015). This latter effect could be explained by the immune suppression induced by the pesticide. Indeed, Di Prisco et al. (2013) reported an inhibition of an immune cascade effector by the neonicotinoid clothianidin, which hinders the bee’s control of *Deformed wing virus* (DWV) replication. We also observed that tolerance to chronic bee paralysis virus (CBPV) infection was changed by co-exposure to thiamethoxam but this effect may depend on the honey bee’s physiology (Coulon et al., 2018, 2019). Most studies were performed in laboratories in controlled conditions. As a result, little is known about their consequences on bee behavior, such as orientation flights and foraging activity. We therefore tested the potential effects of a co-exposure to a pathogen (DWV) and a common pesticide (thiamethoxam) in natural conditions.

*Deformed wing virus* is one of the most prevalent honey bee viruses in the world (Martin and Brettell, 2019); for example, it was detected in 97% of tested French apiaries in 2002 (Tentcheva et al., 2004). It is a single strand positive RNA virus, of the *Picornavirales* order, which contains many of the viruses infecting honey bees (Remnant et al., 2017). However, as for many honey bee viruses, DWV mostly causes covert infections in hives (de Miranda and Genersch, 2010). Overt infections often occur when the virus is transmitted to the pupae by the *Varroa destructor* mite through injection while the mite feeds (de Miranda and Genersch, 2010; Möckel et al., 2011), causing deformed wings. In addition, DWV infection is known to impair associative learning and memory formation (Iqbal and Mueller, 2007), weaken flight ability (Wells et al., 2016), induce precocious foraging trips (Benaets et al., 2017) and drastically reduce bee lifespan (Rueppell et al., 2017). Thiamethoxam is a neonicotinoid insecticide commonly used around the world (Sanchez-Bayo, 2014), especially on rapeseed oil, a crop that is widespread and attractive to honey bees (Simon-Delso et al., 2015). It can be quickly metabolized both in insects and plants into clothianidin (Nauen et al., 2003; Coulon et al., 2018), which is also commercialized as an insecticide. Sublethal doses of thiamethoxam have been shown to cause negative effects on homing flights in foragers (Henry et al., 2012). Such impaired foraging behavior after chronic exposure to sublethal doses of another neonicotinoid (thiachloprid) was correlated with a disruption in learning and memory functions (Tison et al., 2016, 2017). Furthermore thiamethoxam or its metabolite clothianidin have been found to cause a significant reduction in foraging activity and longer foraging trips in exposed foragers (Schneider et al., 2012), impair memory consolidation and retrieval (Tison et al., 2019) and inhibit the honey bee immune system (Brandt et al., 2016) and detoxification genes (Coulon et al., 2019). Altogether, loss of bees and poor performance of foragers may affect the colony homeostasis and development, and ultimately lead to colony failure (Perry et al., 2015; Prado et al., 2019).

To better understand the influence of DWV and thiamethoxam co-exposure on honey bees, we recorded survival and the onset of foraging of bees with optical bee counters (Dussaubat et al., 2013; Alaux et al., 2014; Bordier et al., 2017b; Prado et al., 2019). In parallel we measured variation in viral loads as well as the expression levels of immune, detoxification genes, and vitellogenin, which promotes the longevity of bees by acting as an antioxidant (Seehuus et al., 2006) and has an important role in immune function (Amdam et al., 2004).

## Materials and Methods

### Experimental Set Up

Two 5-frame colonies were selected based on low DWV infection levels (<2.5 × 10^6^ copies DWV/bee) and then equipped with optical counters at the hive entrance in early May 2016 and April 2017. Colonies were treated against varroa at the end of the summer using Apivar (Veto-pharma, Palaiseau, France) and overwintered successfully. They were screened for common viruses (DWV, SBV, BQCV, ABPV, IAPV, CBPV) before starting the experiment by analyzing a pool of 40 honey bees per colony (see below for virus quantification). Emerging bees were collected from brood frames originating from the same “DWV-low” colonies, and incubated overnight at 34°C. Newly emerged bees were randomly distributed in cages and marked with either a 3-mm wide barcode printed on laminated paper and glued (Sader) onto the thorax (66 and 60 bees per treatment, colony and replicate in 2016 and 2017, respectively; see Supplementary Table S1), or a paint mark on the abdomen (one color per treatment, 80–100 bees per treatment and replicate, Supplementary Table S1). In total 3024 bees were barcoded and 3125 painted. After tagging or painting, bees were kept overnight in an incubator at 34°C with saturated humidity and 50% sucrose syrup. On the next day, they were assigned to the following treatments: (1) control bees; (2) bees individually fed with 0.25 ng of thiamethoxam in 5 μl of syrup; (3) bees injected with PBS (Phosphate Buffer Saline solution); (4) bees injected with PBS and individually fed with 0.25 ng of thiamethoxam; (5) bees injected with DWV (∼10^4^ copies/bee), and 6. bees injected with DWV (∼10^4^ copies/bee) and individually fed with 0.25 ng of thiamethoxam. The experiment was replicated five times, in May and July 2016, and three times between April and May 2017. The PBS injection treatments were performed as a control for the effects of the injection, as it has been shown that piercing honey bee cuticle can challenge their immunity (Evans et al., 2006; Siede et al., 2012; Alaux et al., 2014).

In the three replicates from 2017 (Supplementary Table S1), additional experimental groups were included: (7) bees individually fed with an inoculum of ∼10^8^ copies of DWV, (8) bees individually fed with 1.00 ng of thiamethoxam, (9) bees injected with DWV (∼10^4^ copies/bee) and individually fed with 1.00 ng of thiamethoxam, and (10) bees individually fed with ∼10^8^ copies of DWV and with 0.25 ng of thiamethoxam.

### Thiamethoxam and DWV Exposures

To obtain the DWV inoculum, the stored DWV sample already described (KX373899, Dalmon et al., 2017) was injected in pupae for multiplication. After 24 h at 34°C and saturated humidity, five pupae were crushed into 500 μl of PBS and then centrifuged twice for 10 min at 8,000 × *g* and 4°C to eliminate most tissue and cell debris. Bees were injected with supernatant from the second centrifugation, and an aliquot of supernatant was later quantified using real-time RT-PCR to retrospectively assess the exact number of viral copies injected. Dilution was calculated to result in 10^8^ copies of DWV in the 5 μl sucrose syrup for DWV-*per os* or to 2.75 × 10^4^ copies of DWV in the 46 nl of the inoculum that was injected into each bee. No quantifiable levels of BQCV, SBV, CBPV, ABPV, IAPV, and KBV were found. Injections were performed using a Nanoject (Drummond Scientific, Broomall, PA, United States) and heat-elongated glass microcapillary tubes, between the third and fourth tergites of bees previously anesthetized with CO_2_ and maintained on ice. These doses corresponded to DWV levels observed from low to high varroa infestations in the colonies (Traynor et al., 2016).

Exposure to thiamethoxam was performed as follows. After 2 h of starvation (control bees included), bees were individually fed with 5 μl of syrup. The 0.25 and 1.00 ng doses of thiamethoxam in 5 μl of syrup solution were obtained from successive dilutions from a 1 mg/ml solution of thiamethoxam, first in water, then in 30% syrup. Syrup was prepared by mixing 30% w/v powdered sugar in water. The same technique was used to feed the 10^8^ copies/bee of DWV for the *per os* treatments.

After treatments, 396–725 bees per hive were introduced into the hives. For viral and gene expression analysis, three samples of three bees for each replicate and treatment (identified from their paint mark on the abdomen) were collected from colony frames and immediately put on dry ice 24 and 48 h after their re-introduction into the colony, and then stored at −80°C (Supplementary Table S1).

### Onset of Foraging and Survival

In total, 3024 bees tagged with barcodes were followed using optical counters, as previously described in Alaux et al. (2014) and Bordier et al. (2017b). Briefly, the bee counter is composed of a modified entrance with eight tunnels, a camera monitoring the entrance, a computer for image acquisition and software that analyses the images and records the in-and-out activity of bees. For each detection event, we recorded the bee’s ID, direction (in or out of the hive) and the time (day, hour, minute, and second). From these raw data, we retrieved the duration of each flight and identified the first day of foraging for each individual, defined as the age at which each bee performs a trip lasting longer than 10 min (Marco Antonio et al., 2008; Woyciechowski and Moroń, 2009; Benaets et al., 2017). All tagged bees were followed until no bee could be detected (up to 51 days); for each bee the last day of detection was considered as time of death. 2306 out of the 3024 barcoded bees were recorded at least once, corresponding to 76% detection.

### Virus and Gene Transcription Quantification

The number of DWV copies was determined by quantitative PCR using a StepOne-Plus Real-Time PCR System (Applied Biosystems, Life Technologies) and the SYBR Green detection method. Pools of 3 bees were crushed directly in 900 μl of Qiazol with a 0.8 cm-diameter bead using a TissueLyser (Qiagen) (four times 30 s at 30 Hz). The homogenate was centrifuged for 2 min at 12,000 × *g* and 4°C, and the supernatant was transferred into a new tube to be processed for RNA extraction. Then, total RNA was extracted using Qiagen’s RNeasy Universal Plus Mini Kit, following the manufacturer’s instructions (QIAGEN, Hilden, Germany). RNA was quantified using a spectrophotometer (Nanodrop 2000, Thermo Fisher Scientific) and then diluted to obtain a concentration of 500 ng/μl. RNA solutions were stored at −80°C. Reverse transcription was performed from 1 μg RNA with the High capacity RNA to cDNA kit (Applied Biosystems, Saint Aubin, France) according to the manufacturer’s protocol.

For virus quantification, 3 μl of 10-fold diluted cDNA were mixed with 7 μl of SYBR Green master mix (Applied Biosystems) containing 10 pmol of primers. DWV, but also ABPV, CBPV, BQCV and *Sacbrood bee virus* (SBV) loads were quantified using a qPCR absolute quantification. Amplification was performed with the following program: 10 min 95°C, 40 cycles of 15 s at 95°C, and 1 min at 60°C. A melting curve was generated from 60 to 95°C. Quantification was performed twice. Sequence primers and viral sequences used as reference are shown in Supplementary Table S2. A standard curve, obtained for each virus from serial dilutions of viral synthetic fragments of known concentration (MWG, Germany), was used to calculate viral loads from Ct values in the samples (Dalmon et al., 2019).

Expression level of immune response [vitellogenin, dorsal-1-a, apidaecin, pro-phenoloxydase (*ppo*)] and detoxification (glutathione-*S*-transferase 3, catalase and *cyp6as11*) genes were assessed using the primer pairs reported in Supplementary Table S2. We focused on DWV injected bees co-exposed, or not, to thiamethoxam at 0.25 ng, because for coexposure to DWV and 1.00 ng of thiamethoxam, honey bee mortality was too high to allow for sufficient sampling (Supplementary Table S1). Relative gene expression data were analyzed using β-actin and RpL32 and the geometric mean of both as a reference (Reim et al., 2013). The qRT-PCR mix for one sample was prepared according to the recommendations of the Power SYBR Green RNA-to-Ct 1-Step Kit (Thermo Fisher Scientific): 10 μl of RT-PCR SYBR Green mix, 0.2 μl of 10 μM forward and reverse primer each, 0.16 μl of Retro-transcriptase enzyme from the kit, 8.44 μl of H_2_O, and finally 1 μl of RNA sample. All primer pairs were designed using PrimerExpress 3.0 software (Life Technologies) following the standard procedure. Negative (H_2_O) and positive controls (previously identified positive samples) were included in each qRT-PCR run. To ensure that the amplification efficiencies of the target and reference genes were approximately equal, the amplification of five 10-fold dilutions of the total RNA sample (from 1.0 to 0.1 ng per reaction) were analyzed in triplicate. The efficiency plot for Log input total RNA vs. ΔCt had a slope lower than ± 0.1. Amplifications for relative gene expression quantification were performed using the StepOne Real-Time PCR System (Applied Biosystems, Life Technologies) with the following thermal cycling profiles: one cycle at 48°C for 15 min for reverse transcription, one cycle at 95°C for 10 min, 40 cycles at 95°C for 15 s and 60°C for 1 min, and one cycle at 68°C for 7 min.

### Statistics

For statistical analysis, bees exposed to DWV via oral ingestion (*per os*) or injection were analyzed separately. *Per os* treatments included Control, thiamethoxam at 0.25 ng, thiamethoxam at 1.00 ng, DWV *per os* and co-exposure to DWV *per os* and thiamethoxam at 1.00 ng. Injection treatments included PBS injection (control), co-exposure to PBS and thiamethoxam at 0.25 ng (control for injected bees exposed to thiamethoxam), DWV injection, co-exposure to DWV and 0.25 ng of thiamethoxam, and co-exposure to DWV and 1.00 ng of thiamethoxam.

Bee mortality was analyzed with a Kaplan–Meier estimation (Efron, 1988; Pepe and Fleming, 1989) and survival rates were calculated using the Cox proportional hazards model (Cox, 1972). The age at the first foraging flight (first flight longer than 10 min) was determined for each bee. The proportion of bees lost after their first exit from the hive was calculated using the χ^2^ of compliance, and by comparing observed and expected proportions. A χ^2^ table was used first to compare all treatments with 9 degrees of freedom, then separately for each pairwise combination of treatments with 1 degree of freedom. Variations in the age at which honey bees performed their first foraging trip were analyzed via a general linear mixed model fit by maximum likelihood (Laplace Approximation) using the Poisson probability distribution function. Treatment and month (April as basal level) were considered as fixed factors and the source colony as a random factor. The total time spent outside the hive was analyzed via pairwise Wilcoxon rank-sum tests; *p*-values were adjusted using the Holm–Bonferroni method. Flights less than 10 min were included to take into account all flights and not only foraging trips. Variations in the time spent outside were analyzed via a generalized least square model for 1–30 and 6–23 days old bees from the oral inoculation and injection treatments, respectively, to avoid a low sampling bias when mortality increased.

For genes, analyses were carried out on ΔCt values (log_2_ scale). Viral loads were analyzed from the log_10_ of the number of copies per bee. Analyses were carried out using either ANOVA followed by Tukey HSD when data followed a normal distribution (non-significant Shapiro–Wilk test) or a pairwise Wilcoxon test with Bonferroni correction when data did not follow a normal distribution (significant Shapiro–Wilk test). All statistical analyses were run with the software R (Version 1.0.143 – 2009–2016 RStudio).

## Results

### Thiamethoxam Influence on DWV Load in Honey Bees

We assessed whether exposure to DWV, thiamethoxam alone or a combination of both modified the virus levels in experimentally infected bees using pairwise Wilcoxon tests (df = 10, *n* = 228 pools of three bees). While *per os* infection with DWV did not induce a higher level of DWV as compared to control bees (*p* = 1, Supplementary Figure S1), exposure by injection triggered a slight increase of DWV level in bees (*p* = 0.049, *n* = 82). Thiamethoxam exposure did not affect DWV levels; neither did its combination with DWV infection *per os*. A significant increase of DWV loads was observed after DWV injection in bees exposed to 0.25 ng of thiamethoxam (*p* = 0.003, *n* = 30) but was not higher than in DWV injected bees. No increase in DWV level was found in bees co-exposed to DWV injection and 1.00 ng of thiamethoxam, but sampling size was low (only seven samples could be retrieved, Supplementary Table S1).

For all other tested viruses (ABPV, BQCV, CBPV, SBV) no significant variation due to treatments was detected using Wilcoxon pairwise tests with Bonferroni correction, df = 10, *n* = 228 pools of three bees (Supplementary Figure S2).

### Honey Bee Survival and Onset of Foraging Activity

#### Survival

**FIGURE 1:**
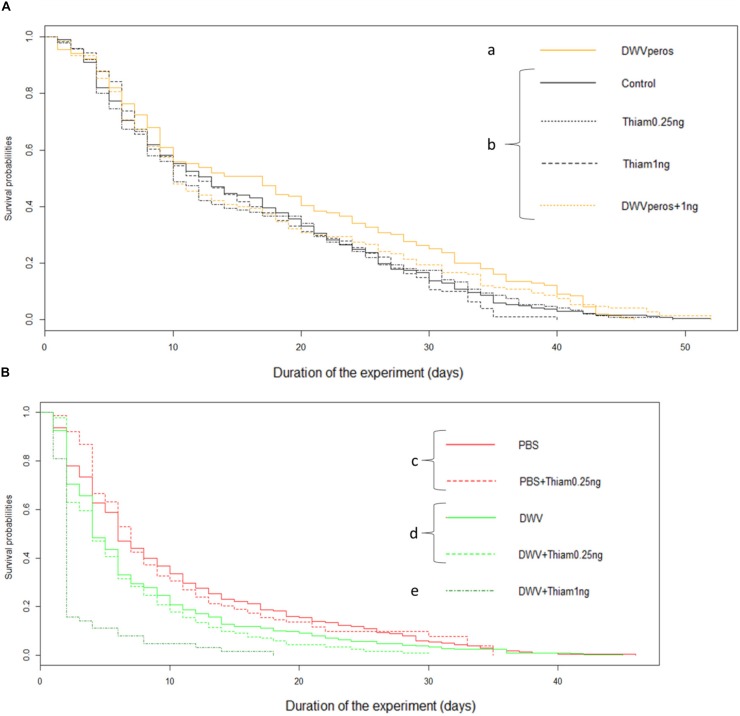
Survival curves of bees exposed to thiamethoxam and/or DWV. While oral exposure to thiamethoxam and/or DWV did not decrease survival compared to the control group, injection of DWV did. A drastic decrease of survival was observed in bees co-exposed to DWV and thiamethoxam 1.00 ng. **(A)** Exposure to pesticide and virus *per os*: untreated bees (Control, *n* = 339 bees), Thiamethoxam at 0.25 ng/bee (Thiam 0.25 ng, *n* = 302), Thiamethoxam at 1.00 ng/bee (Thiam 1 ng, *n* = 150), DWV *per os* (*n* = 153), DWV *per os* co-exposed with Thiamethoxam at 1.00 ng (DWV *per os* + Thiam 1 ng, *n* = 150); **(B)** Exposure to pesticide *per os* and to DWV by injection: bees injected with PBS (PBS, used here as control, *n* = 311 bees), PBS co-exposed with Thiamethoxam at 0.25 ng (PBS + Thiam 0.25 ng, *n* = 209), DWV (*n* = 368), DWV co-exposed with thiamethoxam at 0.25 ng/bee (DWV + Thiam 0.25 ng, *n* = 253), DWV co-exposed with thiamethoxam at 1.00 ng/bee (DWV + Thiam 1 ng, *n* = 63). Different letters show statistical differences (*p* < 0.05) between groups (log-rank test).

Regardless of the dose, thiamethoxam did not affect bee survival when compared to control bees (control: *n* = 338, 0.25 ng: *n* = 305, *p* = 0.384 and 1.00 ng: *n* = 150, *p* = 0.836, Figure 1A), which was expected for these sublethal doses.

Surprisingly, *per os* infection with DWV significantly increased survival probability as compared to control bees (*n* = 156, df = 4, *p* = 0.018). As expected, bee survival strongly decreased after DWV injection (Figure 1B) in comparison to non-injected bees (Figure 1A). Moreover, the injection of DWV significantly increased mortality when compared to bees injected with PBS (PBS control: *n* = 310, injected DWV: *n* = 369, *p* < 0.001, Figure 1B).

Co-exposure to 1.00 ng thiamethoxam and DWV-*per os* (*n* = 150) did not influence bee survival as compared to control bees (*n* = 338) or 1.00 ng thiamethoxam-exposed bees (*n* = 150) (*p* = 0.212 and *p* = 0.276, respectively). The lowest dose of thiamethoxam (0.25 ng) did not affect the survival of DWV- or PBS-injected bees (*n* = 253, *n* = 212, respectively) when compared to bees only exposed to DWV (injected DWV: *n* = 369) or PBS (PBS control: *n* = 310), (df = 4, *p* = 0.122 and *p* = 0.876, respectively). However, exposure to the highest dose of thiamethoxam (1.00 ng) drastically decreased the survival of DWV-injected bees (*n* = 63, df = 4, *p* < 0.001 for all treatment comparisons). In fact, 4 days after the co-exposure, only 10% of bees from this group were alive compared to 66% for DWV-injected bees and 80% for bees exposed to 1.00 ng of thiamethoxam.

#### Lost Bees

**FIGURE 2:**
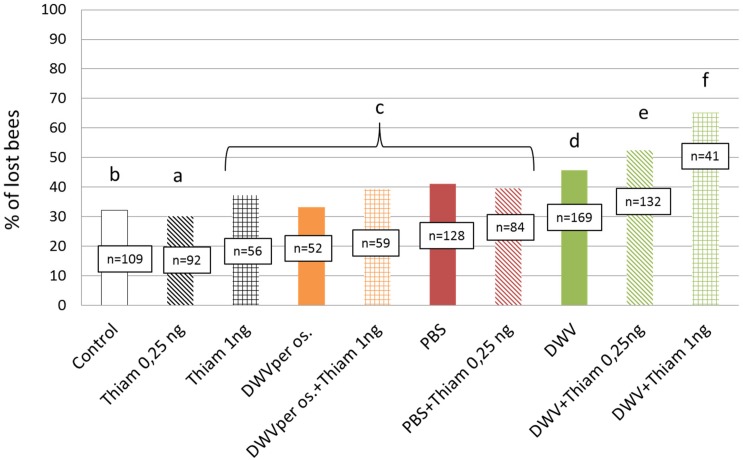
Proportion of bees that were lost after their first exit when exposed to thiamethoxam and/or DWV. Number of lost bees are given for each treatment. DWV injection significantly increased the loss of bees at the first exit; bee losses being higher when co-exposed to thiamethoxam. Treatments are: untreated control bees, bees fed with 0.25 ng of thiamethoxam, bees fed with 1.00 ng of thiamethoxam, bees fed with DWV (*per os*), bees co-exposed to thiamethoxam at 1.00 ng and DWV *per os*, bees injected with PBS (PBS, used here as control), PBS co-exposed with 0.25 ng of thiamethoxam (PBS + Thiam 0.25 ng), DWV, DWV co-exposed with thiamethoxam at 0.25 ng/bee (DWV + Thiam 0.25 ng), DWV co-exposed with thiamethoxam at 1.00 ng/bee (DWV + Thiam 1 ng). Letters show significant differences between groups (*p* < 0.05) from lowest to highest proportion of lost bees (a << f).

The optical counter allowed us to record the number of bees that never returned to the colony after their first flight. The lowest proportion of lost bees was found among bees exposed to 0.25 ng of thiamethoxam (*p* < 0.001 when compared to all others groups, χ^2^ = 20.83, df = 1). Exposure to thiamethoxam at 1.00 ng, DWV *per os*, DWV *per os*/1.00 ng of thiamethoxam, PBS injection, and PBS injection/0.25 ng of thiamethoxam induced significantly higher proportions of lost bees (χ^2^ = 62.33, df = 9, *p* < 0.001) as compared to control bees. Nevertheless, the highest proportions of lost bees were observed in the following groups: DWV injection, DWV injection/0.25 ng of thiamethoxam, and DWV injection/1.00 ng of thiamethoxam (Figure 2). Finally, co-exposure to DWV injection and 1.00 ng of thiamethoxam caused a significantly higher proportion of lost bees than DWV alone (χ^2^ = 21.79, df = 1, *p* < 0.001).

#### Onset of Foraging

The age at onset of foraging differed between replicates (Supplementary Table S3). Indeed, for all treatments, bees started foraging overall significantly earlier in May and July than in April (Supplementary Table S3).

**FIGURE 3:**
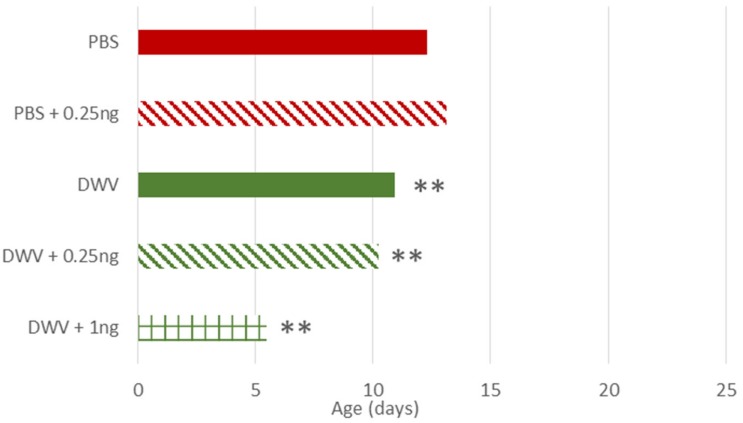
Estimation of age of injected-honey bees at first foraging flight. DWV- injected bees performed their first foraging flight earlier than control bees, as well as bees co-exposed to thiamethoxam 0.25 ng or much thiamethoxam 1.00 ng. Treatments are: bees injected with PBS (PBS, used as control), injected with PBS co-exposed with thiamethoxam at 0.25 ng/bee (PBS + 0.25 ng), injected with DWV (DWV), injected with DWV co-exposed with thiamethoxam at 0.25 ng/bee (DWV + 0.25 ng), injected with DWV co-exposed with thiamethoxam at 1.00 ng/bee (DWV + 1 ng). For oral exposures, see Supplementary Table S3. ** indicates a significant difference (*p* < 0.01) based on the ANOVA performed from the general linear mixed model.

Regarding the treatment effects, among the non-injected bees (*per os*) only bees exposed to 0.25 ng of thiamethoxam exhibited a later onset of foraging (*p* = 0.015), but this difference was only observed in July (Supplementary Table S3). Among the injected bees, DWV, with or without co-exposure to thiamethoxam (0.25 and 1 ng), caused an earlier onset of foraging (at least 1 day earlier) as compared to PBS-injected bees (respectively *p* = 0.002, *p* = 0.004, *p* = 0.007, Figure 3 and Supplementary Table S3). The strongest impact was observed in bees co-exposed to DWV and 1.00 ng of thiamethoxam (Figure 3 and Supplementary Table S3, *n* = 21 surviving bees after their first exit out of 120 treated and 63 detected at least once), with an earlier onset of foraging than bees exposed to DWV alone (*n* = 195) or in combination with 0.25 ng of thiamethoxam (*n* = 119), suggesting a synergistic effect.

#### Daily Flight Duration and Total Time Spent Outside

**FIGURE 4:**
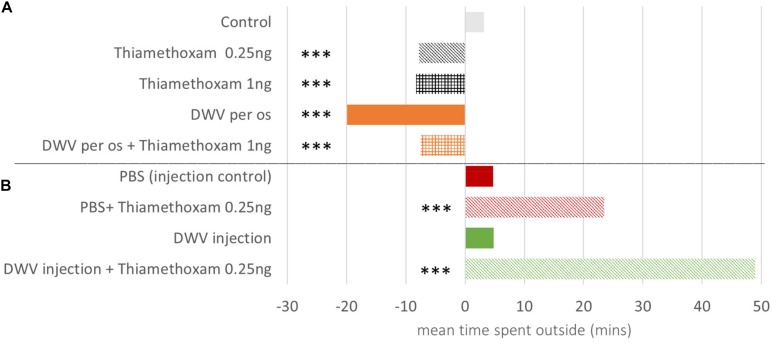
Daily increase in honey bee flight duration as compared to control bees. Honey bees orally exposed to DWV and/or thiamethoxam exhibit a slower daily increase of flight duration compared to control bees (negative values) **(A)**. Bees injected and co-exposed to thiamethoxam 0.25 ng/bee exhibit a faster daily increase of flight duration compared to the injected control bees (PBS) (positive values) **(B)**. *** indicates significant difference (*p* < 0.001) based on ANOVA performed from the general linear mixed model. Variations in the daily time spent outside the hive are considered for 1–30 and 6–23 days old bees from the oral inoculation and injection treatments, respectively, to avoid a low sampling bias when mortality increased. DWV injection + Thiamethoxam 1.00 ng is missing because of the high mortality (>80% on the 6–23 days old bees period, Figure 1). Detailed data are in Supplementary Table S4.

As bees became older their foraging activity increased (around 3.2 and 4.7 min. each day in control bees and PBS injected bees, respectively, Supplementary Table S4). Among oral exposure treatments (df = 12696), from day one to day 30 both doses of thiamethoxam and DWV reduced the daily flight duration as compared to control bees (Figure 4A). Injections (df = 3633), on the contrary, tended to increase the daily flight duration from day 6 to 23 (Figure 4B); this was observed for bees exposed to DWV, 0.25 ng of thiamethoxam, or a combination of both. Due to the early mortality of bees co-exposed to DWV by injection and thiamethoxam at 1.00 ng/bee, this treatment could not be included in this analysis (Figure 1).

**FIGURE 5:**
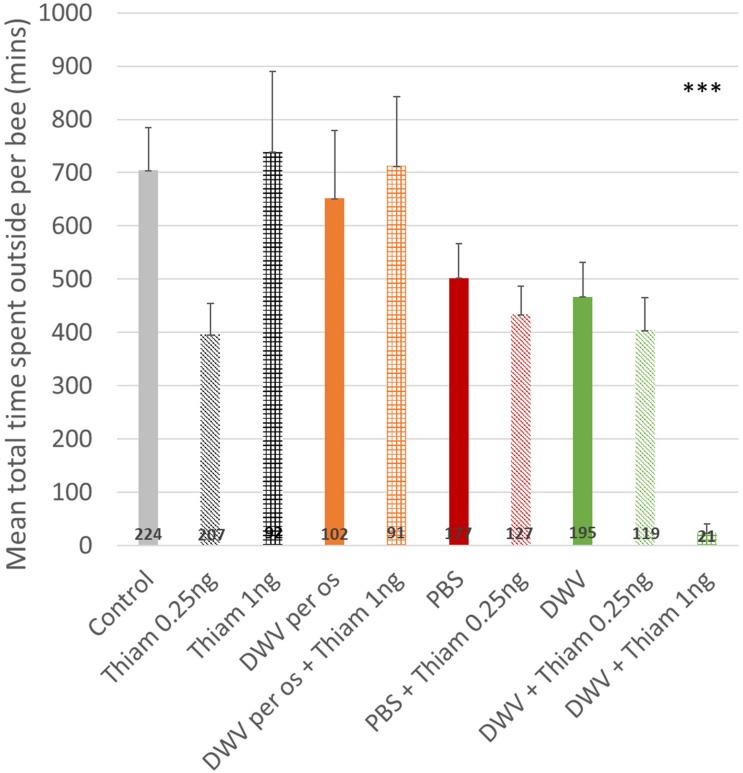
Total time spent outside for honey bees. The total time spent outside for honey bees co-exposed to DWV (injection) and thiamethoxam 1.00 ng was drastically lower (***: *p* < 0.001, Wilcoxon rank sum test). The number of bees per treatment is at the bottom of the bars. Treatments are oral exposure to thiamethoxam at 0.25 ng/bee (Thiam 0.25 ng), thiamethoxam at 1.00 ng/bee (Thiam 1 ng); bees injected with PBS (PBS, used as control), injected with PBS co-exposed with 0.25 ng of thiamethoxam (PBS + Thiam 0.25 ng), injected with DWV (DWV), injected with DWV co-exposed with thiamethoxam at 0.25 ng/bee (DWV + Thiam 0.25 ng), injected with DWV co-exposed with thiamethoxam at 1.00 ng/bee (DWV + Thiam 1 ng).

Total time spent outside was calculated using all exit data, including those shorter than 10 min, from day one until last recorded day. Co-exposure to DWV (injection) and 1 ng of thiamethoxam reduced the time accumulated outside (Figure 5), which was significantly shorter than all other treatments (about 24 min compared to 397–738 min, *p* < 0.001, Figure 5). Interestingly no effect was observed between others treatments, even though DWV and/or thiamethoxam fed groups spent less daily time outside, because they survived for a longer period of time.

#### Expression Level of Immune and Detoxification Genes

Dorsal-1-a, apidaecin, pro-phenoloxydase, glutathione-S-transferase 3, catalase and *cyp6as11* genes did not show any significant variation in their expression level between experimental treatments (*p* > 0.05) (data not shown).

**FIGURE 6:**
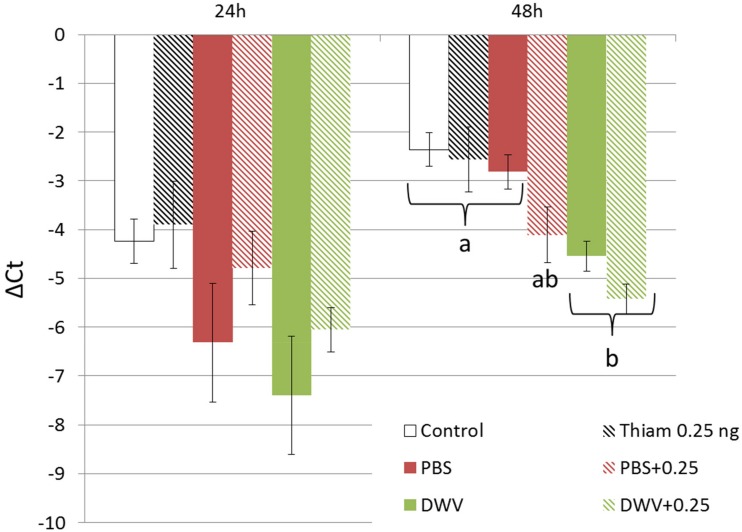
Vitellogenin expression level in bees exposed to DWV and/or thiamethoxam. Mean vitellogenin expression significantly decreased 48 h after exposure to DWV alone (green bars) or in combination with 0.25 ng of thiamethoxam (hatched green bars) compared to control bees, bees exposed to 0.25 ng of thiamethoxam, and PBS-injected bees (injection control). Analyses were performed on bees sampled 24 or 48 h post exposure. Letters indicate significant differences (*p* < 0.05).

No difference in vitellogenin expression levels was found between treatments 24 h post exposure (*p* > 0.05). At 48 h post exposure, vitellogenin expression in control bees was not significantly different between bees injected with PBS (*p* = 0.940), bees exposed to thiamethoxam at 0.25 ng (*p* = 0.999), or bees co-exposed to 0.25 ng of thiamethoxam and PBS injection (*p* = 0.081; Figure 6). However bees injected with DWV or co-exposed to DWV and 0.25 ng of thiamethoxam exhibited significantly lower vitellogenin expression levels than control bees (*p* < 0.001 for both), bees injected with PBS (*p* = 0.011 and *p* < 0.001, respectively), or bees exposed to 0.25 ng of thiamethoxam (*p* = 0.015 and *p* < 0.001, respectively). Vitellogenin expression was not significantly different between bees injected with DWV and bees co-exposed to DWV injection and 0.25 ng of thiamethoxam (*p* = 0.603).

## Discussion

By co-exposing honey bees to DWV and thiamethoxam at environmentally relevant doses in their natural environment (colony), we showed that the combination of both stressors caused premature foraging, a higher rate of failure to return to the colony and a steep decrease in survival.

We demonstrated that honey bees co-exposed to DWV and 1.00 ng of thiamethoxam exhibited the lowest survival rate. Interestingly, exposure to thiamethoxam (0.25 ng or 1.00 ng) as a single stressor did not affect bee survival at all, but mortality was increased in bees injected with DWV alone or co-exposed to DWV and 0.25 ng of thiamethoxam. These results correspond with previous studies showing that DWV has a deleterious effect on honey bee lifespan (Woyciechowski and Moroń, 2009; Dainat et al., 2012; Martin et al., 2013; Benaets et al., 2017; Rueppell et al., 2017). Notably, by using a Radio-Frequency Identification device, Benaets et al. (2017) found that experimentally DWV infected bees had a greater early-life mortality (up to 81%) as compared to control bees (up to 67%), and Martin et al. (2013) observed a high decrease in longevity of adults that were parasitized by varroa during the larvae/pupae stages. In our study, no high increase of DWV loads was observed in virus-injected bees, which is congruent with the absence of variation of several immune genes (dorsal-1-a, apidaecin, pro-phenoloxydase) and with the native background of DWV infection. The injection of DWV did increase DWV loads in the bees and viral loads tend to be higher when they were co-exposed with 1.00 ng of thiamethoxam. However, the mortality due to co-exposure to DWV and the neonicotinoid was so high that sampling size decreased dramatically compared to the other treatments, resulting in a low number of replicates. Most of the infected bees may have been excluded by nest mates as part of social immunity (Baracchi et al., 2012; McDonnell et al., 2013), or may have altruistically removed themselves (Rueppell et al., 2010) or simply died and could not be sampled. Indeed, during the experiments, some co-exposed bees were removed by workers from the colony while still alive. In a previous *in vitro* study, bees co-exposed to CBPV and thiamethoxam (5.00 ng/bee) showed higher CBPV loads in dead bees, as opposed to live bees (Coulon et al., 2018), which suggests that higher DWV loads may be present in dead bees, but could not be measured, as bees did not return or were excluded from the hive.

We did not find any significant effect of *per os* DWV inoculation on viral loads, even when we performed exposure with higher numbers of DWV copies (inoculum with 10^8^ copies of DWV per bee). Ryabov et al. (2014) showed that larvae reared in DWV symptomatic hives (thus exposed through alimentation), but not infested by *Varroa*, showed low DWV loads close to control (larvae raised in colonies with low DWV levels and no *Varroa*), while *Varroa* parasitized pupae developed very high loads of DWV (direct *Varroa* transmission). Feeding does not seem to be an efficient way of experimental transmission of DWV in honey bees (Bailey and Ball, 1991). However, we measured viral loads 48 h after virus feeding and we cannot exclude that DWV loads may have increased later. The infection may have been delayed due to the degradation of viral particles in the gut and may be first restricted to the midgut epithelium before breaching this barrier (Möckel et al., 2011). The absence of a significant effect on the number of lost bees, onset of foraging and flight behavior is, however, congruent with an inefficient transmission of DWV *per os*. Surprisingly, survival was slightly higher when feeding the bees with DWV extracts. These extracts originated from pupae ground in buffer and sequentially centrifuged, and may contain associated beneficial microbiota, which could enhance bee survival (Raymann et al., 2017). Moreover, the ingested DWV could have triggered non-specific defense mechanisms preventing its multiplication (Evans and Spivak, 2010). The lower daily flight duration we observed in DWV orally exposed bees may also explain why they outlived control bees. Behavioral maturation may have been delayed, thus increasing bee survival (Perry et al., 2015).

The survival decline in bees co-exposed to DWV and 1.00 ng of thiamethoxam was related to a very high proportion of bees that never returned to the colony after their first exit (65%). One simple explanation is that co-exposed bees got lost on their return trips, which has been observed for foragers after a similar dose of thiamethoxam (Henry et al., 2012). However, contrary to Henry et al. (2012), we did not find a significant loss in bees only exposed to thiamethoxam. The main difference is that honey bees in Henry et al. (2012) were released 1 km away from their colonies, while in our study, bees were not exposed to this return flight challenge. Moreover, our trial required us to anesthetize and/or starve bees, which were also kept for some hours in the lab before re-introducing them into the colonies. This experimental procedure may have stressed bees and increased overall mortality.

The lowest survival rate and higher rate of lost bees in the DWV injected and 1.00 ng thiamethoxam co-exposed group could be explained by their early onset of foraging. Indeed, such young honey bees may not be optimally adapted to foraging tasks and are usually less resilient than normal-aged foragers (Woyciechowski and Moroń, 2009). Young workers are heavier and exhibit lower flight performance (Vance et al., 2009), likely due to different flight metabolic rates and muscle biochemistry (Schippers et al., 2006). Precocious foraging has been previously observed from DWV injections by Benaets et al. (2017) in the same range (2.3 days earlier vs. 2.4 days earlier in our study), but also with other stressors, such as *Varroa destructor* (Downey et al., 2000) and *Nosema apis* (Wang and Mofller, 1970). Considering that there is no effect of thiamethoxam alone, and the fact that honey bees that were co-exposed to DWV and thiamethoxam started foraging earlier (2–5 days earlier depending on thiamethoxam dose) our results suggest that the effect of co-exposure between DWV injection and thiamethoxam on this specific trait could be synergistic.

Virus and pesticide treatments could affect physiological traits of bees. To further explore the influence of both DWV and thiamethoxam on bees, we analyzed potential physiological changes, by focusing on immune and detoxification genes. Except for vitellogenin, none of the tested genes were affected by the treatments, suggesting a lack of effect on the immune and detoxification system in our experiments (at least 24 and 48 h post treatments). This contradicts previous *in vitro* studies, which, for example, showed that immune genes under control of *dorsal-1a (Apidaecin)* were down-regulated by sublethal doses of clothianidin (Di Prisco et al., 2013); or that dorsal-1a was down-regulated in bees with high levels of DWV in the presence of Varroa (Nazzi et al., 2012) or exposed to CBPV and/or thiamethoxam (Coulon et al., 2019). Up-regulation of some immune genes in bees with high levels of DWV in natural conditions has already been observed (Steinmann et al., 2015) and illustrates that gene expression, and especially immune response, varies greatly (Doublet et al., 2017) and depends on the experimental conditions and time of sampling from exposure, and may be tissue-specific (here, whole bee bodies were analyzed). Moreover, we cannot exclude that these genes may have been down- or up-regulated earlier or later after co-exposure.

However we noticed a significant decrease of vitellogenin expression level. Vitellogenin is involved in the regulation of division of labor, notably a key player in the regulation of behavioral maturation, with a higher expression in nurses than in foragers (Nelson et al., 2007; Marco Antonio et al., 2008). The maintenance of stable conditions within colonies relies on a division of labor, with bees spending the first 2–3 weeks of their adult life working in the hive (feeding and taking care of the brood, building comb), and then the rest of their life outside of the hive (foraging for nectar and pollen to supply the colony growth) (Seeley, 1995). However, a significant loss of foragers for the colony will accelerate the behavioral maturation of young bees to replace them (Robinson, 1992). Similarly, parasitism also leads to precocious foragers (Dussaubat et al., 2013; Goblirsch et al., 2013; Natsopoulou et al., 2015), which may not be optimally adapted to foraging tasks (Schippers et al., 2006; Vance et al., 2009). The down-regulation of vitellogenin we observed here upon DWV injection is consistent with previous studies performed on other stress factors such as simulated heat waves (Bordier et al., 2017a). Nelson et al. (2007) and Marco Antonio et al. (2008) showed that an RNAi-mediated inhibition of the transcription of vitellogenin made bees start foraging significantly earlier. We therefore propose that injection of DWV, reinforced by co-exposure to thiamethoxam, induced a precocious shift toward foraging activity through down-regulation of the transcription of vitellogenin. Here co-exposed bees may become precocious, but immature, foragers that could induce a high cost on the hive for collecting nectar and pollen resources. Depending on the number of affected bees, this could result in a breakdown of division of labor, as indicated by Perry et al. (2015).

## Conclusion

Our results underscore the importance of translating laboratory experiments to the field. By performing behavioral experiments, we could identify highly negative effects of pesticide/virus co-exposure on honey bee survival and identify some underlying mechanisms (early onset of foraging, lost bees and flight duration). The relationship between survival and flight behavior could not be assessed from previous cage experiments (Di Prisco et al., 2013; Coulon et al., 2018, 2019). Our results provide new insights on the negative synergism of viruses (DWV) and pesticides (neonicotinoids) on bees, even at sublethal environmental pesticide doses. Such a great impact on honey bee survival and flight behavior was quite unexpected from a single acute oral exposure to a sublethal dose of thiamethoxam. In the field, honey bees may be exposed to higher thiamethoxam doses (from 13.3 ng/g in nectar to 20.2 ng/g in honey, 53.3 ng/g in stored pollen or 86 ng/g in pollen from field margin plants; Mullin et al., 2010; Bargañska et al., 2013; Botías et al., 2015) and may not be exposed only once but probably several times while foraging and/or feeding on honey and stored pollen. As co-exposure to both viruses and pesticides likely occurs in natural conditions, field relevant experiments are essential to better understand the influence of these stress factors in bees and the underlying mechanisms potentially leading to colony failure.

## Data Availability Statement

All datasets generated for this study are included in the article/Supplementary Material.

## Author Contributions

AD, MC, YL, and GD conceptualized and designed the experiments. ED, AP, MR-C, RT, CA, and AP provided conceptualization advice. MC and AD performed the experiments with assistance from FA. AD, GD, and YL provided lab facilities and resources for experiments. AD and YL supervised the research. MC, AP, and AD did the statistical analysis. MC and AD drafted the manuscript. All authors participated in the revisions of the manuscript, read and approved the final manuscript.

## Conflict of Interest

The authors declare that the research was conducted in the absence of any commercial or financial relationships that could be construed as a potential conflict of interest.
