# Supplementary material for: Interactions Between Thiamethoxam and Deformed Wing Virus Can Drastically Impair Flight Behavior of Honey Bees
Source: Front Microbiol. 2020 Apr 30;11:766. doi: 10.3389/fmicb.2020.00766 (PMC7203464; doi:10.3389/fmicb.2020.00766)
Supplement: Supplementary file 1 [file Table_1.pdf]

Setting				Treatments									
Experiment	Replicate	Month	Year	Control	Thiam 0.25 ng	Thiam 1.00 ng	DWV *per os*	DWV *per os* Thiam 1.00 ng	PBS	PBS+Thiam 0.25 ng	DWV	DWV+Thiam 0.25 ng	DWV+Thiam 1.00 ng
Optical counters	1	05/05/2016	2016	132 (66x2 colonies)	132 (66x2 colonies)	na	na	na	132 (66x2 colonies)	132 (66x2 colonies)	132 (66x2 colonies)	132 (66x2 colonies)	na
	2	10/07/2016	2016	132 (66x2 colonies)	132 (66x2 colonies)	na	na	na	132 (66x2 colonies)	132 (66x2 colonies)	132 (66x2 colonies)	132 (66x2 colonies)	na
	3	12/04/2017	2017	60	na	60	60	60	60	na	60	na	na
	4	27/04/2017	2017	60	60	60	60	60	60	na	60	60	60
	5	16/05/2017	2017	60	60	60	60	60	60	na	60	60	60
Total number of bees detected at least once				338	305	150	156	150	310	212	369	253	63
Painted bees	1	05/05/2016	2016	100	100	na	na	na	100	100	100	100	na
	2	10/07/2016	2016	100	100	na	na	na	100	100	100	100	na
	3	12/04/2017	2017	80	na	80	80	80	80	na	80	na	na
	4	27/04/2017	2017	80	80	80	80	80	83	na	79	80	83
	5	16/05/2017	2017	80	80	80	80	80	80	na	80	80	80

Table S1: Sampling size for survival and onset of foraging experiment (bee counter).
