# Supplementary material for: Interactions Between Thiamethoxam and Deformed Wing Virus Can Drastically Impair Flight Behavior of Honey Bees
Source: Front Microbiol. 2020 Apr 30;11:766. doi: 10.3389/fmicb.2020.00766 (PMC7203464; doi:10.3389/fmicb.2020.00766)
Supplement: Supplementary file 2 [file Table_2.pdf]

Name	Sequence	bp	Reference
CYP6AS14<br>F<br>R	 TGACATTGAGTTGACGGACGAT<br>GAAACCTGCCGCGAAGAA	64	this work
GSTS3<br>F<br>R	 AAACCGATAGCGCAGAGTAACG<br>CATCATTGCCTCCCATTCGT	87	this work
Catalase<br>F<br>R	 TTTGGTGGGCCTAGAGAATGTC<br>TCCTCCTTTGGGTCTACATCATAAC	92	this work
Apidaecin<br>F<br>R	 TTTTGCCTTAGCAATTCTTGTTG<br>GTAGGTCGAGTAGGCGGATCT	81	Di Prisco et al. 2016
Dorsal-1A<br>F<br>R	 TCGGATGGTGCTACGAGCGA<br>AGCATGCTTCTCAGCTTCTGCCT	153	Di Prisco et al. 2016
Vitellogenin<br>F<br>R	 AACGCCGTGAAGGTGAACAG<br>TATCGTAGAGAACCTCGCATTTCC	109	this work
PPOAct<br>F<br>R	 CGTTGAAAAGTCGAAGCAGATTAA<br>AGGACGCCACCGCAGTATT	112	this work
β-actin<br>F<br>R	 GATTTGTATGCCAACACTGTCCTT<br>TTGCATTCTATCTGCGATTCCA	69	Di Prisco et al. 2016
RpL32<br>F<br>R	 CGTCATATGTTGCCAACTGGTTT<br>CCATGAGCAATTTCAGCACAA	107	this work
DWV<br>DWV-F8668<br>DWV-B8757	 TTCATTAAAGCCACCTGGAACATC<br>TTTCCTCATTAACTGTGTCGTTGA	136	Locke et al. 2012
BQCV<br>BQCV-F7893	 AGTGGCGGAGATGTATGC	294	Locke et al. 2012

BQCV-B8150	GGAGGTGAAGTGGCTATATC		
CBPV<br>CBPV-F1818<br>CBPV-B2077	 CAACCTGCCTCAACACAG<br>AATCTGGCAAGGTTGACTGG	296	Locke et al. 2012
SBV<br>SBV-F3164<br>SBV-B3461	 TTGGAACTACGCATTCTCTG<br>GCTCTAACCTCGCATCAAC	226	Locke et al. 2012
ABPV<br>ABPV-F6548<br>KIABPV-B6707	 TCATACCTGCCGATCAAG<br>CTGAATAATACTGTGCGTATC	197	Locke et al. 2012

Table S2: Primers used for the quantification of selected honey bee genes and viruses.

Locke, B., et al. (2012). "Host adaptations reduce the reproductive success of Varroa destructor in two distinct European honey bee populations." Ecology and Evolution 2(6): 1144-1150.

Di Prisco, G., et al. (2016). "A mutualistic symbiosis between a parasitic mite and a pathogenic virus undermines honey bee immunity and health." Proceedings of the National Academy of Sciences 113(12): 3203-3208.
