# Supplementary material for: Interactions Between Thiamethoxam and Deformed Wing Virus Can Drastically Impair Flight Behavior of Honey Bees
Source: Front Microbiol. 2020 Apr 30;11:766. doi: 10.3389/fmicb.2020.00766 (PMC7203464; doi:10.3389/fmicb.2020.00766)
Supplement: Supplementary file 3 [file Table_3.pdf]

Dependant variable	Covariate	Class	Coefficient	s.e.m.	P-value	Corresponding mean age (days)
Age at first foraging flight (>10min) - oral exposures	Intercept	Control	3.083	0.145	**<0.001**	**21.83**
	Treatments	DWV *per os*	0.021	0.042	0.625	22.29
		Thiamethoxam 0.25 ng/bee	0.086	0.035	**0.015**	**23.78**
		Thiamethoxam 1 ng/bee	-0.072	0.044	0.104	20.32
		DWV *per os* + thiamethoxam 1 ng	-0.066	0.045	0.144	20.45
	Replicates	May	-0.624	0.162	**<0.001**	**11.70**
		July	-0.632	0.179	**<0.001**	**11.61**
Age at first foraging flight (>10min) - injections	Intercept	PBS	2.510	0.088	**<0.001**	**12.30**
	Treatments	PBS+Thiam 0.25 ng	0.066	0.052	0.198	13.14
		DWV	-0.120	0.045	**0.007**	**10.91**
		DWV+Thiam 0.25 ng	-0.181	0.059	**0.001**	**10.26**
		DWV+Thiam 1ng	-0.806	0.289	**0.004**	**5.50**
	Replicates	May	-0.333	0.098	**<0.001**	**8.82**
		July	-0.544	0.108	**<0.001**	**7.14**

Table S3: Coefficients (estimates), standard errors and associated *P*-values for the selected models investigating a treatment effect on bee age at first foraging trips. Mean age of first foraging trip is calculated as $e^{(coefficient)}$. Predicted mean ages of first flight are bold when statistically different from controls (Control or PBS injection).
