# Supplementary material for: Interactions Between Thiamethoxam and Deformed Wing Virus Can Drastically Impair Flight Behavior of Honey Bees
Source: Front Microbiol. 2020 Apr 30;11:766. doi: 10.3389/fmicb.2020.00766 (PMC7203464; doi:10.3389/fmicb.2020.00766)
Supplement: Supplementary file 4 [file Table_4.pdf]

Dependant variable	Covariates	Class	Value	s.e.m	*P*-value
Mean daily increase in flight duration (minutes) - oral exposures	Intercept (Age)	Control	3.248	0.085	<0.001
	Treatments	Thiam 0.25ng	-7.785	1.747	<0.001
		Thiam 1ng	-8.291	2.188	<0.001
		DWV *per os*	-19.952	2.065	<0.001
		DWV *per os* +0.25ng	-7.503	2.216	<0.001
Mean daily increase in flight duration (minutes) - injections	Intercept (Age)	PBS	4.720	0.297	<0.001
	Treatments	PBS+0.25ng	23.502	3.623	<0.001
		DWV	4.836	2.123	0.0228
		DWV+0.25ng	49.063	4.501	<0.001

Table S4: Mean daily increase in flight duration compared to intercept (Age x Control treatment). Standard errors and associated *P*-values for the selected models investigating an effect of the treatments on flight duration are also shown.
