# Supplementary material for: Interactions Between Thiamethoxam and Deformed Wing Virus Can Drastically Impair Flight Behavior of Honey Bees
Source: Front Microbiol. 2020 Apr 30;11:766. doi: 10.3389/fmicb.2020.00766 (PMC7203464; doi:10.3389/fmicb.2020.00766)
Supplement: Supplementary file 5 [file Image_1.pdf]

Interactions between thiamethoxam and *Deformed wing virus* can drastically impair flight behaviour of honey bees

M. Coulon[1,2]†, A. Dalmon[1]†[*], G. Di Prisco[3,4], A. Prado[1, 5], F. Arban[1], E. Dubois[2], M. Ribière-Chabert[2], C. Alaux[1], R. Thiéry[2], Y. Le-Conte[1].

[1]INRAE, UR 406 Abeilles et Environnement, Site Agroparc, F84914 Avignon, France

[2]ANSES Sophia Antipolis, Unit of Honey bee Pathology, 105, route des Chappes, F06902 Sophia Antipolis, France

[3]CREA-AA, Council for Agricultural Research and Economics, Agriculture and Environment Research Centre, Bologna, Italy

[4]University of Napoli "Federico II", Department of Agriculture, Portici, Napoli, Italy

[5]Escuela Nacional de Estudios Superiores Juriquilla, UNAM, Querétaro 76230, México

*** Correspondence:**
anne.dalmon@inrae.fr

†These authors have contributed equally to this work

Supplementary Material

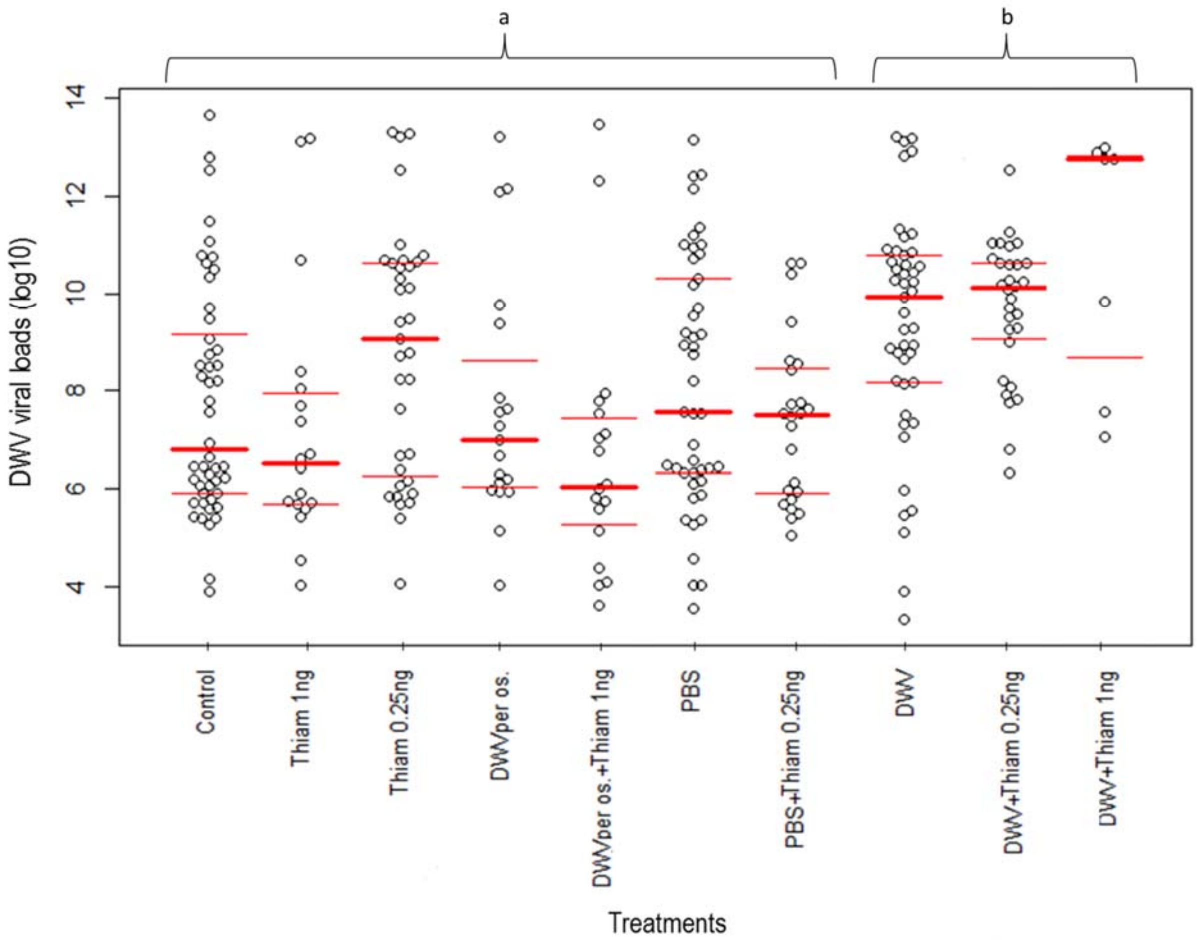

Figure S1: DWV infection level in bees exposed to thiamethoxam and/or DWV. Each dot represents the DWV viral load in one sample of 3 pooled bees. The different treatments are: control bees, bees injected with PBS (PBS), bees injected with PBS and exposed to 0.25 ng of thiamethoxam (PBS+0.25ng), bees exposed to 0.25 or 1.00 ng of thiamethoxam (Thiam 0.25 ng and Thiam 1ng, respectively), bees infected with DWV (DWV *per os* and DWV injection, respectively), and bees co-exposed to DWV and thiamethoxam (DWV *per os* and Thiam 1ng, DWV and Thiam 0.25ng, DWV and Thiam 1ng). Viral loads are shown in $\log_{10}$. As in standard boxplots, red lines represent the first quartile (25%), the median (50%) and the third quartile (75%). Different letters show statistical differences between groups.
