# Supplementary material for: Interactions Between Thiamethoxam and Deformed Wing Virus Can Drastically Impair Flight Behavior of Honey Bees
Source: Front Microbiol. 2020 Apr 30;11:766. doi: 10.3389/fmicb.2020.00766 (PMC7203464; doi:10.3389/fmicb.2020.00766)
Supplement: Supplementary file 6 [file Image_2.pdf]

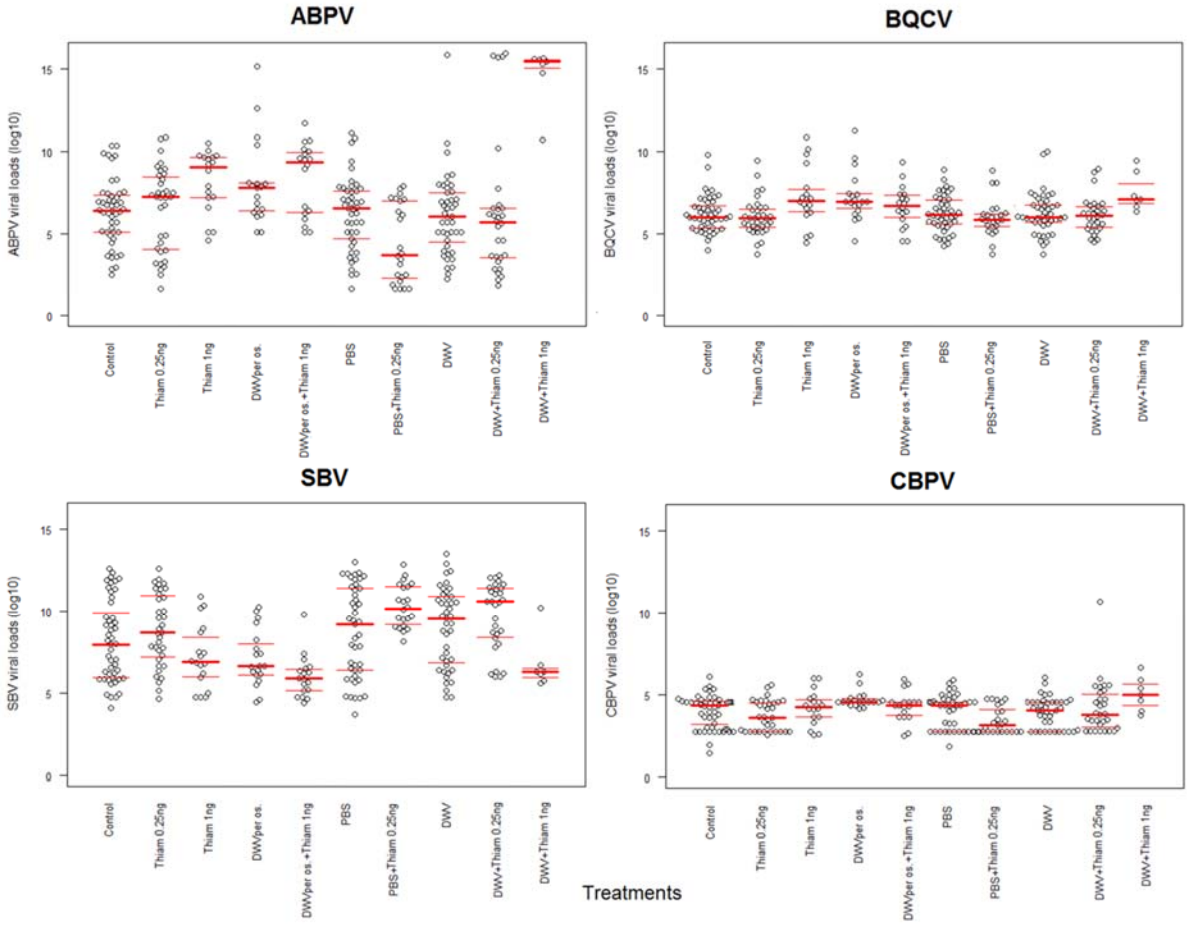

Figure S2: Viral load distributions for the four additionally tested viruses.

Viral loads are shown in $\log_{10}$. No significant variation in viral loads was detected between treatments (Wilcoxon tests, df=10, n= 228 pools of 3 bees). The different treatments are: control bees, bees injected with PBS (PBS), bees injected with PBS and exposed to 0.25 ng of thiamethoxam (PBS+0.25ng), bees exposed to 0.25 or 1.00 ng of thiamethoxam (Thiam 0.25ng and Thiam 1ng, respectively), bees infected with DWV (DWV *per os* and DWV injection, respectively), and bees co-exposed to DWV and thiamethoxam (DWV *per os* and Thiam 1ng, DWV and Thiam 0.25ng, DWV and Thiam 1ng). In boxplots, red lines represent the first quartile (25%), the median (50%) and the third quartile (75%). Swarms show the distribution of populations, representing each sample.
